# Синдром множественных эндокринных неоплазий 1 типа: анализ данных 102 пациентов из 43-х семей в популяции Российской Федерации

**DOI:** 10.14341/probl13642

**Published:** 2026-03-07

**Authors:** Р. Х. Салимханов, А. К. Еремкина, Х. В. Багирова, К. Мейрамбек, С. В. Попов, Н. Г. Мокрышева

**Affiliations:** Национальный медицинский исследовательский центр эндокринологии им. академика И.И. ДедоваРоссия; Endocrinology Research CentreRussian Federation

**Keywords:** синдром множественных эндокринных неоплазий 1 типа, первичный гиперпаратиреоз, генотип-фенотипическая корреляция, нейроэндокринные неоплазии, multiple endocrine neoplasia syndrome type 1, primary hyperparathyroidism, genotype-phenotypic correlation, neuroendocrine neoplasia

## Abstract

**ОБОСНОВАНИЕ:**

ОБОСНОВАНИЕ. Синдром множественных эндокринных неоплазий 1 типа (МЭН-1) — редкое аутосомно-доминантное заболевание, обусловленное инактивирующими мутациями в гене MEN1. МЭН-1 характеризуется высокой пенетрантностью; к «классической триаде» проявлений синдрома относятся первичный гиперпаратиреоз (ПГПТ), нейроэндокринные неоплазии (НЭН) желудочно-кишечного тракта (ЖКТ) и аденомы гипофиза. Диагноз устанавливается на основании клинических, семейных и генетических критериев, однако фенотипическая вариабельность и отсутствие четкой генотип-фенотипической корреляции затрудняют раннюю диагностику заболевания.

**ЦЕЛЬ:**

ЦЕЛЬ. Охарактеризовать клинико-эпидемиологические, лабораторно-инструментальные и генетические особенности семейных форм МЭН-1 в Российский Федерации.

**МАТЕРИАЛЫ И МЕТОДЫ:**

МАТЕРИАЛЫ И МЕТОДЫ. На базе ФГБУ «НМИЦ эндокринологии им. академика И.И. Дедова» Минздрава России проведено одноцентровое одномоментное исследование (102 пациента с генетически верифицированным МЭН-1 из 43 семей) за период 2015–2025 гг. Пациенты были разделены 3 группы в зависимости от возраста манифестации ПГПТ — в большинстве случаев «первого» компонента заболевания: ≤18 лет, 19–40 лет и >40 лет. Кроме того, все пациенты были сгруппированы в зависимости от типа и локализации мутации в гене MEN1. Проведен сравнительный анализ независимых групп и подгрупп по основным показателям кальций-фосфорного обмена, характеристикам проявлений, течению и исходам хирургического лечения ПГПТ, проанализирована генотип-фенотипическая корреляция МЭН-1.

**РЕЗУЛЬТАТЫ:**

РЕЗУЛЬТАТЫ. Группы пациентов с МЭН-1 были сопоставимы по основным характеристикам (пол, первый компонент, другие проявления синдрома; p>0,05), однако выявлены значимые различия в возрасте манифестации НЭН (p<0,001) и тенденция для аденом гипофиза (p=0,003). При анализе генетических данных мы обнаружили ассоциацию между мутацией в 10-м экзоне гена MEN1 и риском развития аденом гипофиза (ОР=7,7; p=0,006), в то время как тип мутации никак не определял фенотип МЭН-1. Группы не различались по множественному характеру поражения околощитовидных желез (ОЩЖ), хирургическим исходам ПГПТ, а также гистологическим характеристикам образований ОЩЖ (p>0,05).

**ЗАКЛЮЧЕНИЕ:**

ЗАКЛЮЧЕНИЕ. Анализ семейных форм МЭН-1 в российской популяции подтвердил высокую клиническую гетерогенность синдрома, включая ассоциацию мутаций в 10-м экзоне гена MEN1 с повышенным риском аденом гипофиза. У пациентов с манифестацией ПГПТ в более молодом возрасте отмечалось более раннее развитие НЭН ЖКТ и аденом гипофиза. Результаты исследования подтверждают необходимость раннего генетического скрининга и индивидуального мониторинга пациентов с МЭН-1, а также их родственников.

## Abstract (en)

**BACKGROUND:**

BACKGROUND: Multiple endocrine neoplasia syndrome type 1 (MEN-1) is a rare, autosomal dominant disorder resulting from inactivating mutations in the MEN1 gene. It demonstrates high penetrance, with the "classic triad" of manifestations comprising primary hyperparathyroidism (PHPT), gastrointestinal neuroendocrine neoplasms (NEN), and pituitary adenomas. Diagnosis relies on clinical, familial, and genetic criteria. However, significant phenotypic variability and the lack of a clear genotype-phenotype correlation complicate early diagnosis.

**AIM:**

AIM: To investigate the clinical-epidemiological, laboratory-instrumental, and genetic characteristics of familial MEN-1 in the Russian population.

**MATERIALS AND METHODS:**

MATERIALS AND METHODS: We conducted a single-center, single-stage study (2015–2025) at the Endocrinology Research Centre enrolling 102 genetically confirmed MEN-1 patients from 43 families. Participants were stratified by age at PHPT onset (≤18 years, 19–40 years, and >40 years) and MEN1 mutation type/location. We compared groups using calcium-phosphorus metabolism parameters, disease progression patterns, PHPT surgical outcomes, and genotype-phenotype correlations.

**RESULTS:**

RESULTS: Cohort baseline characteristics (sex, manifestations) were comparable (p>0,05), but we observed significant differences in NEN onset age (p<0,001) and a trend toward higher pituitary adenoma prevalence (p=0,003). Exon 10 mutation were associated with a 7,7-fold increased pituitary adenoma risk (OR=7,7; p=0,006), though mutation type did not predict broader phenotype. Groups did not differ in multiglandular involvement, surgical outcomes, or histopathology (p>0,05).

**CONCLUSION:**

CONCLUSION: MEN-1 exhibits marked clinical heterogeneity in the Russian population, with exon 10 mutations significantly increasing pituitary adenoma risk. Early-onset PHPT predicts earlier NEN and pituitary adenoma development. These findings support proactive genetic screening and risk-stratified monitoring for MEN-1 families.

## Обоснование

Синдром множественных эндокринных неоплазий 1 типа (МЭН-1, OMIM #131100), также известный как синдром Вернера, представляет собой редкое аутосомно-доминантно наследуемое заболевание (распространенность 3–20/100 000 человек), характеризующееся активацией различных путей туморогенеза в органах эндокринной и других систем. МЭН-1 обусловлен инактивирующими мутациями в гене MEN1, локализующемся на хромосоме 11q13 и кодирующем белок менин — регулятор клеточного цикла и апоптоза [1]. Синдром характеризуется высокой пенетрантностью (до 95% к 50 годам), отсутствием явных генотип-фенотипических корреляций [2][3]. Основные компоненты МЭН-1 включают первичный гиперпаратиреоз (ПГПТ), нейроэндокринные новообразования (НЭН) желудочно-кишечного тракта (ЖКТ) и гипофиза [4]. Согласно европейским клиническим рекомендациям, диагноз «МЭН-1» устанавливается на основании следующих критериев: клинического — два или более МЭН-1-ассоциированных образований из «классической триады»; семейного — один из компонентов синдрома и наличие мутации в гене MEN1 у родственника первой линии родства; генетического — подтвержденная инактивирующая гетерозиготная мутация в гене MEN1 [1].

Фенотипическая вариабельность синдрома существенно затрудняет его раннюю диагностику и прогнозирование течения. Скрининг родственников внутри семей с МЭН-1 необходим для раннего распознавания и динамического контроля заболевания. Современные методы молекулярно-генетических исследований позволяют не только верифицировать диагноз, но и осуществить предиктивное тестирование членов семьи, что в свою очередь благоприятно сказывается на прогнозе и качестве жизни пациентов [5].

Примерно у 5–25% пациентов с клиническими проявлениями МЭН-1 по результатам генетического тестирования отсутствует мутация в гене MEN1 — такие случаи классифицируются как «фенокопии» синдрома [6]. Кроме того, МЭН-1-подобный фенотип может быть обусловлен мутациями в других генах, например, CDKN1B, CDKN2B или CDKN2C [7]. В отличие от МЭН-1, у пациентов с мутацией в гене CDKN1B ниже риск НЭН поджелудочной железы, что стало дополнительным основанием для выделения отдельной нозологии — МЭН-4 [7].

Настоящее исследование представляет собой первый крупный анализ семейных случаев МЭН-1 в Российской Федерации (РФ). Мы сфокусировались на клинических, лабораторно-инструментальных характеристиках пациентов, а также эпидемиологических и генетических аспектах синдрома. Понимание паттернов течения МЭН-1 позволит улучшить стратегии наблюдения и своевременного вмешательства у пациентов и их близких.

## Цель исследования

Охарактеризовать клинико-эпидемиологические, лабораторно-инструментальные и генетические особенности семейных форм МЭН-1 в РФ.

## Материалы и методы

Согласно поставленной цели, в период с 01.10.2015 по 01.01.2025 гг. на базе на базе ФГБУ «НМИЦ эндокринологии им. академика И.И. Дедова» Минздрава России (далее — НМИЦ эндокринологии) было проведено одноцентровое одномоментное исследование. В рамках работы 102 пациентам было выполнено генетическое исследование (преимущественно — в НМИЦ эндокринологии), по результатам которого верифицирована мутация в гене MEN1. Анализ таргетной генетической панели, включающей кодирующие области 378 генов, связанных с эндокринопатиями (в том числе MEN1, RET, CDKN1B, CASR, CDC73), проведен 12,7% пациентам (n=13); секвенирование по Сэнгеру — 81,4% (n=83), у 5,9% (n=6) информация о методе генетического исследования не была доступна. Массивное параллельное секвенирование проводилось на платформе Illumina NextSeq 550 (Illumina, США); секвенирование по Сэнгеру осуществлялось с использованием генетического анализатора AB3500 (Thermo Fisher Scientific, США). Основаниями для назначения генетического исследования являлись подтвержденный ПГПТ у пациентов моложе 40 лет и/или сочетание двух и более компонентов синдрома МЭН-1 и/или рецидив/персистенция ПГПТ после хирургического лечения и/или наличие кровных родственников с синдромом МЭН-1. Все пациенты, включенные в представленное исследование, проходили либо амбулаторное, либо стационарное обследование и лечение в НМИЦ эндокринологии, однако у части обследования на момент манифестации заболевания могли быть выполнены в других медицинских учреждениях, а в карту внесены лишь их результаты. Все пациенты имели отягощенный по МЭН-1 семейный анамнез (т.е., как минимум, одного кровного родственника с подтвержденной мутацией в гене MEN1). Период активного наблюдения за пациентами составил 1–10 лет, у 19,4% (n=20) пациентов данных за манифестацию любого из компонентов МЭН-1 получено не было в связи с недоступностью результатов комплексного скрининга.

При анализе истории болезни пациентов учитывались следующие параметры: показатели кальций-фосфорного обмена (концентрации ПТГ, кальция (Сa) общего, ионизированного и альбумин-скорректированного (рассчитан по формуле: альбумин-скорректированный Ca (ммоль/л) = Ca общий (ммоль/л) + 0,02 × [ 40 – альбумин (г/л)]), альбумина, фосфора крови; креатинин с расчетом скорости клубочковой фильтрации (р. СКФ) (СКФ рассчитывалась по формуле CKD-EPI для пациентов 18 лет и старше, по формуле Шварца — младше 18 лет); суточная кальциурия на момент манифестации заболевания до проведения первого хирургического лечения (х/о) ПГПТ); результаты методов топической диагностики ПГПТ (ультразвуковое исследование (УЗИ), сцинтиграфия (⁹⁹mTc-Технетрил) ОЩЖ с ОФЭКТ-КТ, мультиспиральная компьютерная томография (МСКТ) органов шеи и верхнего средостения с контрастным усилением (к/у) в различных комбинациях); наличие висцеральных и костных осложнений ПГПТ, другой МЭН-1-ассоциированной эндокринной патологии. Низкоэнергетические переломы крупных костей скелета (плечевых, бедренных, позвонков грудного и поясничного отделов позвоночника) диагностировались на основании результатов рентгенографии; оценка минеральной плотности костной ткани (МПК) проводилась с помощью двухэнергетической рентгеновской денситометрии (DXA) в поясничном отделе позвоночника, проксимальном отделе бедренной и дистальном отделе лучевой костей. Значения МПК интерпретировались по Т-критерию у мужчин старше 50 лет, женщин в менопаузе, по Z-критерию — у остальных. Осложнения со стороны почек (нефрокальциноз и/или нефролитиаз) устанавливались по данным УЗИ либо МСКТ почек, а также по уровню рСКФ. Эрозивно-язвенная патология ЖКТ оценивалась по результатам эзофагогастродуоденоскопии. Учитывались исходы хирургического лечения — ремиссия/рецидив/персистенция. Диагностика НЭН ЖКТ проводилась по результатам МСКТ с к/у или магнитно-резонансной томографии (МРТ) органов брюшной полости и забрюшинного пространства с к/у, аденомы гипофиза — по результатам МРТ головного мозга с к/у.

Статистический анализ проводился с помощью пакета прикладных программ Statistica v. 13.3 (TIBCO Software Inc., США). Сравнительный анализ трех независимых исследуемых групп по количественным признакам проведен с помощью критерия Краскела-Уоллиса с дальнейшим post-hoc анализом в случае наличия статистически значимых различий. Сравнительный анализ двух независимых групп по количественным признакам проведен с помощью критерия Манна-Уитни. Сравнение независимых групп по качественным признакам проводили с помощью двухстороннего точного критерия Фишера. Уровень значимости (р) при проверке статистических гипотез принимался равным 0,05. Для коррекции критического уровня значимости при множественных сравнениях применялась поправка Бонферрони (р0), после чего значения р в диапазоне между рассчитанным р0 и 0,05 интерпретировались как статистическая тенденция. Исследования с участием людей были рассмотрены и одобрены Комитетом по этике (протокол № 8 от 24.06.2015 г.). Письменное информированное согласие на участие в данном исследовании было предоставлено всеми участниками.

Работа выполнена с использованием материалов Уникальной научной установки «Коллекция биологического материала пациентов с эндокринными патологиями» ФГБУ «НМИЦ эндокринологии им. академика И.И. Дедова» Минздрава России (Москва, Россия).

## Результаты

## Характеристика участников исследования

В исследование вошли 102 пациента из 43 семей (из 21 региона РФ, рис. 1) с верифицированной мутацией в гене MEN1, мужчины/женщины = 1/1,4. МЭН-1 манифестировал с ПГПТ — в 52,4% (n=43), НЭН ЖКТ — в 19,5% (n=16), аденомы гипофиза — в 18,3% (n=15) случаев, бессимптомное носительство мутации в гене MEN1 подтверждено у 9,8% (n=8) пациентов. Среди пациентов с доступными данными ПГПТ был диагностирован у 86,9% (n=73/84), аденомы гипофиза — у 51,9% (n=42/81), НЭН ЖКТ — у 76,8% (n=63/82), НЭН тимуса — у 2,4% (n=2/82), образования надпочечников — у 19,5% (n=16/82), высокодифференцированный рак щитовидной железы — у 3,6% (n=3/83).

**Figure:**
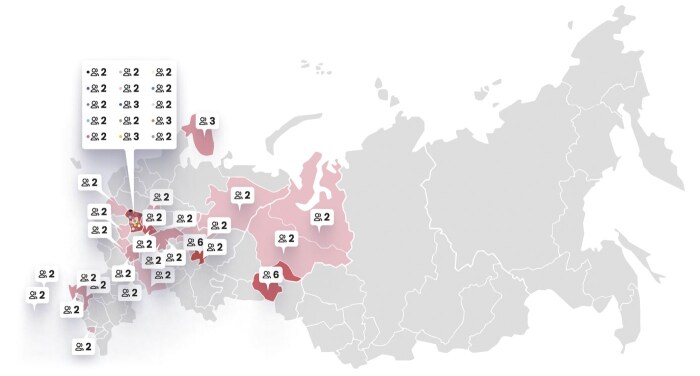
Рисунок 1. Распределение семей пациентов с МЭН-1 в зависимости от регионов РФ.

Указанные пациенты были разделены на 3 группы в зависимости от возраста манифестации ПГПТ, в большинстве случаев — первого компонента заболевания, ≤18 лет, 19–40 лет и >40 лет. Возраст «40 лет» выбран с учетом утвержденных в РФ рекомендаций по скринингу наследственных форм ПГПТ у пациентов моложе указанного возраста. Сравнительные характеристики основных компонентов МЭН-1 представлены в таблице 1.

**Table:** Таблица 1. Сравнительный анализ основных компонентов МЭН-1 у пациентов в зависимости от возраста манифестации ПГПТ ¹критерий Фримена-Холтона *поправка Бонферрони p0=0,05/50=0,0010

Показатель:	n	≤18 лет,Me [ Q1; Q3]/n (%)	n	19–40 лет,Me [ Q1; Q3]/n (%)	n	>40 лет,Me [ Q1; Q3]/n (%)	p-value*
Мужчины	10	5 (50,0%)	41	14 (34,1%)	22	6 (27,3%)	p¹=0,4770
Женщины	5 (50,0%)	27 (65,9%)	16 (72,7%)
Первый компонент МЭН-1 при манифестации (ПГПТ, НЭН, аденома гипофиза)
ПГПТ	10	8 (80,0%)	41	20 (48,8%)	22	15 (68,2%)	p¹=0,0640
НЭН ЖКТ	0 (0,0%)	10 (24,4%)	6 (27,3%)
Аденома гипофиза	2 (20,0%)	11 (26,8%)	1 (4,5%)

Пациенты во всех группах были сопоставимы по полу, первому компоненту МЭН-1, а также по частоте ПГПТ, НЭН ЖКТ, аденом гипофиза (p>0,0500 для всех).

Мы провели сравнительный анализ групп пациентов с МЭН-1 по показателям кальций-фосфорного обмена, а также наличию/отсутствию осложнений ПГПТ (висцеральных: снижение фильтрационной функции почек, структурная патология почек = нефрокальциноз/нефролитиаз, эрозивно-язвенная патология верхних отделов ЖКТ; костных: остеопороз, низкоэнергетические компрессионные переломы позвоночника и внепозвоночные переломы) (табл. 2).

**Table:** Таблица 2. Сравнительный анализ показателей кальций-фосфорного обмена и частоты висцеральных и костных осложнений у пациентов с МЭН-1 в зависимости от возраста манифестации ПГПТ ¹ критерий Фримена-Холтона² критерий Краскела-Уоллиса* поправка Бонферрони p0=0,05/50=0,0010

Показатель:	n	≤18 лет,Me [ Q1; Q3]/n (%)	n	19–40 лет,Me [ Q1; Q3]/n (%)	n	>40 лет,Me [ Q1; Q3]/n (%)	p-value*
Первый визит пациента (перед хирургическим лечением ПГПТ)
ПТГ, пг/мл	10	144,0[ 78,0; 157,0]	39	162,0[ 111,6; 227,0]	20	205,25[ 150,95; 333,95]	p²=0,0229
Ca ионизир., ммоль/л	6	1,37 [ 1,33; 1,42]	22	1,495[ 1,330; 1,580]	10	1,49[ 1,38; 1,55]	p²=0,2178
Ca скор., ммоль/л	3	2,56[ 2,55; 2,80]	18	2,638[ 2,586; 2,980]	13	2,826 [ 2,680; 2,970]	p²=0,1250
P, ммоль/л	7	0,98[ 0,88; 1,08]	25	0,80[ 0,64; 0,92]	16	0,80[ 0,75; 0,91]	p²=0,0330
Ca сут. мочи, ммоль/сут.	5	5,58 [ 4,35; 7,02]	21	8,19[ 7,10; 10,53]	13	10,21[ 9,27; 11,19]	p²=0,0907
25(OH)витамин D, нг/мл	3	23,2[ 13,3; 29,8]	22	24,55[ 16,00; 33,90]	10	21,085[ 13,530; 25,200]	p²=0,4592
Осложнения ПГПТ (перед хирургическим лечением)
рСКФ, мл/мин/1,73 м²	5	103,0[ 80,0; 118,0]	21	108,0[ 95,0; 111,0]	15	92,0[ 73,0; 105,0]	p²=0,1375
Структурная патология почек	9	3 (33,3%)	32	24 (75,0%)	20	12 (60,0%)	p¹=0,0760
Остеопороз	8	1 (12,5%)	29	11 (37,9%)	19	13 (68,4%)	p¹=0,0190
Внепозвоночные низкоэнергетические переломы	9	0 (0,0%)	35	0 (0,0%)	21	1 (4,8%)	p¹=0,4620
Компрессионные переломы тел позвонков (Rg)	-	-	7	1 (14,3%)	9	3 (33,3%)	p¹=0,5850
Эрозивно-язвенная патология ЖКТ	1	0 (0,0%)	21	10 (47,6%)	14	8 (57,1%)	p¹=0,7330

В зависимости от возраста манифестации ПГПТ у пациентов с МЭН-1 нами продемонстрированы различия на уровне статистической тенденции по концентрации ПТГ (p=0,023) и фосфора (p=0,033) крови, при этом группы оставались сопоставимыми по кальциемии, суточной кальциурии и концентрации 25(ОН)витамина D крови (p>0,05 для всех). Среди пациентов нарушение фильтрационной функции почек (рСКФ менее 60 мл/мин/1,73 м²) фиксировалось в 7,3% (n=3) случаев, структурная патология почек — в 63,9% (n=39), из которых нефролитиаз составил 94,9% (n=37), нефрокальциноз — 3,3% (n=2). Остеопороз был диагностирован почти у половины пациентов — 44,6% (n=23), низкоэнергетические переломы: тел позвонков — у 25% (n=4), внепозвоночные переломы крупных костей — у 1,5% (n=1). Эрозивно-язвенная патология верхних отделов ЖКТ встречалась в 50% доступных наблюдений (n=18). Мы выявили различия в группах на уровне статистической тенденции в зависимости от наличия остеопороза (p=0,019). По другим параметрам (функциональная и структурная патология почек; маркеры костного ремоделирования: щелочная фосфатаза, остеокальцин, C-концевой телопептид коллагена I типа; низкоэнергетические позвоночные и внепозвоночные переломы крупных костей; МПК в поясничном отделе позвоночника (L1-4), бедренной (Femur Neck, Total Hip) и лучевой (Radius 33%, Radius Total) костей; эрозивно-язвенная патология верхних отделов ЖКТ) группы были сопоставимы (p>0,05 для всех).

Эктопированные ОЩЖ наблюдались всего в 4,3% случаев (n=3), при этом множественное поражение ОЩЖ, верифицированное гистологическим исследованием, отмечено у 83,3% (n=50) пациентов с ПГПТ. Распределение по морфологическому типу образований ОЩЖ оказалось следующим: аденома — 53,7% (n=29), гиперплазия — 24,0% (n=13), атипическая опухоль — 1,9% (n=1), карцинома — 3,7% (n=2), сочетание аденомы и гиперплазии — 13,0% (n=7), аденомы и атипической опухоли — 3,7% (n=2). Большинству пациентов хирургическое лечение ПГПТ было проведено в объеме субтотальной паратиреоидэктомии — 37,3% (n=22), тотальной паратиреоидэктомии — 22,0% (n=13), удаления одного или двух образований ОЩЖ — 22,0% (n=13) и 18,6% (n=11) соответственно. Трансплантация ОЩЖ в предплечье применялась в 13,1% (n=8) случаев. В послеоперационном периоде хронический гипопаратиреоз (ГипоПТ) развился у 10,2% пациентов (n=6). Распределение по исходам первого хирургического лечения ПГПТ: ремиссия — 58,3% (n=35), рецидив — 23,3% (n=14), персистенция — 18,4% (n=11). Повторная паратиреоидэктомия на момент анализа данных была выполнена 30,9% (n=17) пациентам.

Между группами не было выявлено статистически значимых различий по частоте эктопированных и множественных образований ОЩЖ, а также их гистологическому типу (p>0,05 для всех). Исходы первого хирургического лечения ПГПТ также были идентичными (p>0,05).

Нами был выполнен сравнительный анализ групп пациентов с МЭН-1 по характеристикам аденом гипофиза (табл. 3).

**Table:** Таблица 3. Сравнительный анализ характеристик аденом гипофиза у пациентов с МЭН-1 в зависимости от возраста манифестации ПГПТ ¹ критерий Фримена-Холтона² критерий Краскела-Уоллиса* поправка Бонферрони p0=0,05/50=0,0010

Показатель:	n	≤18 лет,Me [ Q1; Q3]/n (%)	n	19-40 лет,Me [ Q1; Q3]/n (%)	n	>40 лет,Me [ Q1; Q3]/n (%)	p-value*
Аденома гипофиза	10	4 (40,0%)	40	26 (65,0%)	22	11 (50,0%)	p¹=0,2670
Возраст диагностики аденомы гипофиза, лет	3	13,0[ 10,0; 22,0]	26	33,5[ 24,0; 40,0]	10	50,0[ 41,0; 56,0]	p²=0,0030
Акромегалия	2	0 (0,0%)	15	1 (6,7%)	4	1 (25,0%)	p¹=0,5560
Болезнь Иценко-Кушинга	1 (50,0%)	0 (0,0%)	0 (0,0%)
Пролактинома	1 (50,0%)	12 (80,0%)	3 (75,0%)
Соматопролактинома	0 (0,0%)	2 (13,3%)	0 (0,0%)
Микроаденома гипофиза	4	3 (75,0%)	24	18 (75,0%)	11	8 (72,7%)	p¹=1,0000
Макроаденома гипофиза	1 (25,0%)	6 (25,0%)	3 (27,3%)

Группы на уровне статистической тенденции различались по возрасту возникновения аденом гипофиза (p=0,003). При этом соотношение гормонально-активных и -неактивных аденом гипофиза, типы гормональной активности аденом гипофиза оставались идентичными (p>0,05 для всех). 9,5% (n=4/42) пациентов перенесли хирургическое лечение (трансназальная транссфеноидальная аденомэктомия), в трех случаях по поводу резистентных к агонистам дофаминовых рецепторов пролактином и в одном в связи с соматопролактиномой.

Исследуемые группы статистически значимо различались по возрасту возникновения НЭН ЖКТ (p<0,001), соотношение гормонально-активных и -неактивных НЭН ЖКТ, а также их множественный характер были сопоставимы (p>0,05 для всех, табл. 4). Среди гормонально-активных НЭН преобладали инсулиномы — 20,6% (n=13/63), реже встречались гастриномы — 3,2% (n=2/63), у одного из пациентов описана инсулин- и панкреатический полипептид-продуцирующая НЭН — 1,6% (n=1/63). Хирургическое лечение по поводу НЭН ЖКТ было проведено 45% пациентам (n=28/62), аналоги соматостатиновых рецепторов длительного действия получали 11,6% (n=7/60), динамическое наблюдение без терапии применялось у 40% (n=24/60), в остальных случаях использовалась комбинация различных методов лечения.

**Table:** Таблица 4. Сравнительный анализ НЭН ЖКТ у пациентов с МЭН-1 в зависимости от возраста манифестации ПГПТ ¹ критерий Фримена-Холтона² критерий Краскела-Уоллиса* поправка Бонферрони p0=0,05/50=0,0010

Показатель:	n	≤18 лет,Me [ Q1; Q3]/n (%)	n	19–40 лет,Me [ Q1; Q3]/n (%)	n	>40 лет,Me [ Q1; Q3]/n (%)	p-value*	Post-HOC
Гормонально-неактивные НЭН ЖКТ	10	6 (60,0%)	40	28 (70,0%)	22	18 (81,8%)	p¹=0,3780	
Гастринома	10	0 (0,0%)	40	2 (5,0%)	22	0 (0,0%)	p¹=0,6560
Инсулинома	10	0 (0,0%)	40	10 (25,0%)	22	3 (13,6%)	p¹=0,1950
Возраст диагностики НЭН, лет	6	18,5[ 18,0; 19,0]	35	35,0[ 28,0; 39,0]	21	50,0[ 43,0; 58,0]	p²=0,0001	p1/2 = 0,0018 p2/3 = 0,0001 p1/3 = 0,0025
Множественные НЭН	6	3 (50,0%)	34	26 (76,5%)	21	19 (90,5%)	p¹=0,0900	
Хирургическое лечение НЭН	6	0 (0,0%)	35	20 (57,1%)	21	8 (38,1%)	p¹=0,0220
Хромогранин А, нмоль/л	5	2,4[ 0,9; 2,9]	29	1,5[ 1,0; 4,8]	18	3,2[ 1,4; 6,7]	p²=0,4571
Гастрин, пг/мл	5	45,0[ 19,7; 57,8]	22	48,1[ 24,5; 96,7]	18	87,85[ 50,60; 173,00]	p²=0,0460

При сравнении групп нами выявлена статистическая тенденция по частоте развития образований надпочечников (p=0,021), но не по возрасту их манифестации (p>0,05). Всего у одного пациента было диагностировано кортизол-продуцирующее образование надпочечника, 5,8% (n=1/17), данных за другие типы секреции не получено (табл. 5).

**Table:** Таблица 5. Характеристики образований надпочечников у пациентов с МЭН-1 в зависимости от возраста манифестации ПГПТ ¹ критерий Фримена-Холтона² критерий Краскела-Уоллиса* поправка Бонферрони p0=0,05/50=0,0010

Показатель:	n	≤18 лет,Me [ Q1; Q3]/n (%)	n	19-40 лет,Me [ Q1; Q3]/n (%)	n	>40 лет,Me [ Q1; Q3]/n (%)	p-value*
Образование надпочечника	10	0 (0,0%)	40	7 (17,5%)	22	9 (40,9%)	p¹=0,0210
Возраст диагностики образования надпочечника, лет	0	-	7	35,0[ 28,0; 42,0]	10	51,0[ 47,0; 59,0]	p²=1,0000

На следующем этапе работы были проанализировали основные характеристики мутаций в обследованной группе. Большинство из них затрагивали различные экзоны (2-10) гена MEN1 (97,7%, n=42) и только 2,3% (n=1) интрон 8. Распределение мутаций в зависимости от затрагиваемого экзона представлено на рисунке 2. Наиболее часто изменения касались 2, 9 и 10 экзона. Наиболее частыми типами мутаций гена MEN1 были: миссенс — 25,6% (n=11), нонсенс — 23,3% (n=10), дупликации со сдвигом рамки считывания — 13,9% (n=6), делеции со сдвигом рамки считывания — 16,2% (n=7) (рис. 3).

**Figure:**
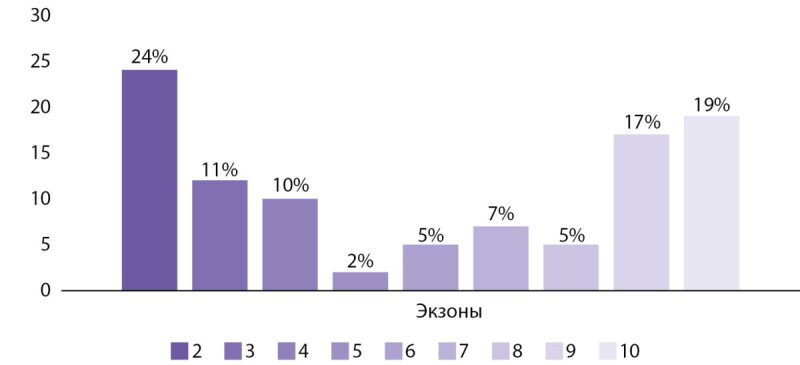
Рисунок 2. Распределение мутаций в гене MEN1 в зависимости от вовлеченного экзона среди пациентов с семейной формой МЭН-1.

**Figure:**
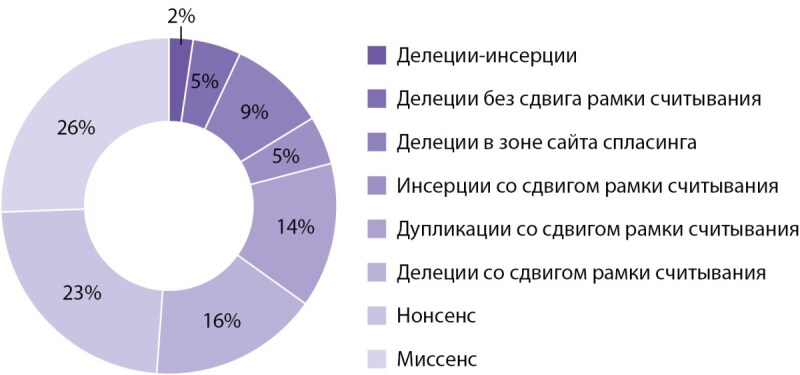
Рисунок 3. Распределение типов мутаций в гене MEN1 среди пациентов с семейной формой МЭН-1.

Пациенты были разделены на 9 групп в зависимости от вовлеченного экзона. Мы не обнаружили никаких значимых различий в фенотипе (возраст манифестации, риск развития основных компонентов заболевания, множественный характер образований ОЩЖ и НЭН ЖКТ, рецидив/персистенция ПГПТ после первого хирургического лечения) пациентов МЭН-1 в зависимости от типа мутации (миссенс, делеции без сдвига рамки считывания vs. нонсенс, инсерции, дупликации и делеции со сдвигом рамки считывания, делеции в области сайта сплайсинга) (p>0,05 для всех). Тем не менее была выявлена статистическая тенденция между наличием мутации в 10 экзоне гена MEN1 и риском развития аденомы гипофиза (p=0,009, критерий Фримана-Холтона), тенденция сохранилась после поправки Бонферрони (p0 с учетом поправки Бонферрони=0,05/9=0,006). Относительный риск (ОР) развития аденомы гипофиза у носителей мутации в 10 экзоне гена MEN1 составил 7,7. Внутри каждой семьи наблюдалась высокая вариабельность компонентов МЭН-1.

## Обсуждение

Большинство случаев МЭН-1 классифицируются как семейные (до 90%) и диагностируются у лиц, имеющих, как минимум, одного родственника первой линии с одним и более основными компонентами заболевания и/или доказанной герминативной мутацией в гене MEN1. У оставшихся 10% пациентов мутация в гене MEN1 возникает de novo без отягощенного наследственного анамнеза [8]. Ключевое значение для выявления семейных форм МЭН-1 имеет ранняя диагностика пробандов (индексных случаев) с последующим обследованием родственников. Отсроченная постановка диагноза МЭН-1 ассоциирована с худшим прогнозом. Так, в недавнем исследовании медианное время между постановкой диагноза МЭН-1 у индексных пациентов и проведением генетического исследования у членов их семьи составило 3,5 года, в течение которых у 20% пациентов развилось метастатическое поражение вследствие НЭН ЖКТ [9]. Проявления МЭН-1 могут наблюдаться с первых лет жизни, поэтому генетическое тестирование у бессимптомных пациентов оправдано с детского возраста [10].

МЭН-1 — аутосомно-доминантно наследуемый синдром, в нашем исследовании незначительно преобладали пациенты женского пола (58,3 против 41,7%), что наблюдалось и при анализе других популяций (Франция, Нидерланды, Италия и Япония) [11–14]. В практически половине случаев (52,4%) МЭН-1 манифестировал с ПГПТ, мы подтвердили данные, полученные ранее в Японии [11] и Италии, другие компоненты синдрома встречались реже (НЭН — в 19,5% случаев, аденома гипофиза — в 18,3%). Среди НЭН ЖКТ в российской популяции существенно чаще диагностировались гормонально-неактивные — 64,6%; как и при анализе баз данных пациентов с МЭН-1 из Японии, США и стран западной Европы, наиболее распространенным типом гормонально-активных НЭН ЖКТ были инсулиномы — 20,6%, реже встречались гастриномы — 3,2%, другой тип гормональной активности (инсулин- и панкреатический полипептид-продуцирующая НЭН) наблюдался всего у одного (1,6%) пациента. В РФ из гормонально-активных аденом гипофиза чаще были диагностированы пролактиномы — 39% пациентов, превышая среднюю распространенность в мире (16–30%) [13][15]. В обследованной популяции при манифестации ПГПТ в молодом возрасте статистически значимо раньше развивались НЭН ЖКТ (p<0,001) и на уровне тенденции — аденома гипофиза (p=0,003). Полученные результаты можно объяснить активным скринингом других компонентов МЭН-1 у пациентов после выявления одного из них.

К настоящему времени идентифицировано более 1500 герминативных и соматических мутацией в гене MEN1 [16][17]. Выявленные нами варианты затрагивали все кодирующие экзоны гена MEN1 (2–10), а также интрон 8. Как и при анализе других баз данных пациентов с МЭН-1 мутации преимущественно локализовались в 2, 9 и 10 экзонах, что может быть объяснено их пропорционально большим размером [18–21]. В то же время потенциальное вовлечение других экзонов/интронов определяет необходимость применения полного секвенирования гена MEN1 в диагностике заболевания. Наиболее частыми типами мутации были: инсерции/делеции/дупликации со сдвигом рамки считывания (34,7%), миссенс (25,6%), нонсенс (23,3%), что в целом согласуется с результатами, опубликованными в мире на других популяциях [19]. В обследованной популяции крупные делеции, затрагивающие более чем 1 экзон гена MEN1, выявлены не были.

Мы не выявили статистически значимых корреляций между типом мутации в гене MEN1 и возрастом манифестации, риском развития основных компонентов заболевания, множественным характером образований ОЩЖ и НЭН ЖКТ, рецидивом/персистенцией ПГПТ после первого хирургического лечения.

Ранее описывалась связь между более высокой частотой НЭН ЖКТ у пациентов с нонсенс-мутациями в гене MEN1 (72,46%) в сравнении с миссенс (54,32%, p=0,022) и мутациями со сдвигом рамки считывания (51,85%, p=0,004) [17], однако авторы признают, что результаты могли носить случайный характер из-за особенностей группы. На примере французской популяции пациентов с МЭН-1 продемонстрирована ассоциация между типом мутации в гене MEN1 и ранней манифестацией ряда компонентов заболевания. Так, крупные перестройки (варианты, приводящие к сдвигу рамки считывания, затрагивающие сайт сплайсинга или нонсенс) способствовали развитию ПГПТ и аденомы гипофиза, но не НЭН ЖКТ в более молодом возрасте [19]. У пациентов с вариантами гена MEN1, укорачивающими белок, и, как следствие, существенно нарушающими его функцию, первый компонент МЭН-1 возникал раньше, при этом возраст диагностики каждого из «классических» проявлений не различался [19]. Согласно анализу флорентийской базы данных пациентов с МЭН-1, НЭН ЖКТ встречались статистически чаще у пациентов с мутациями со сдвигом рамки считывания в сравнении с миссенс-мутациями (68,09% против 43,24%, p=0,022), в то же время ассоциаций между нонсенс- и миссенс-мутациями не было (66,67% против 43,24%, p=0,103), что может указывать на случайную статистическую взаимосвязь [21].

Наше исследование, как и большинство других [21], подтверждает отсутствие достоверной связи между типом мутации в гене MEN1, ее локализацией с особенностями клинических проявлений МЭН-1. Анализ клинических фенотипов пациентов с МЭН-1 из 43-х семей показал высокую вариабельность проявлений заболевания, в том числе среди членов каждой семьи при наличии идентичной мутации. Интересно, что прослеживалась статистическая тенденция между мутацией в 10 экзоне гена MEN1 и развитием у пациентов аденом гипофиза, риск был выше в 7,7 раза выше, чем при мутациях в других частях гена. Уточнение значимости выявленных особенностей станет возможным после проведения более крупных исследований, включающих различные популяции пациентов с МЭН-1.

В настоящее время отмечается переход к более раннему обследованию родственников индексных пациентов с МЭН-1, что является следствием доступности генетического исследования, повышения информированности пациентов о заболевании, а также совершенствования методов диагностического скрининга компонентов синдрома.

## Ограничения исследования

Ввиду различного объема хирургического лечения, возможна погрешность в анализе частоты возникновения послеоперационного гипопаратиреоза и рецидива/персистенции ПГПТ. Кроме того, ограничением представленного исследования является недоступность у части пациентов данных по некоторым анализируемым параметрам, а также проведение лабораторных и/или инструментальных исследований в других медицинских центрах.

## Заключение

Анализ семейных форм МЭН-1 в популяции РФ подтвердил высокую клиническую гетерогенность синдрома, включая вариабельность возраста манифестации и спектра компонентов заболевания. Мы выявили ассоциацию между мутацией в 10 экзоне гена MEN1 и повышенным риском развития аденом гипофиза (ОР=7,7), при этом других значимых корреляций между типом мутации и фенотипическими особенностями МЭН-1 не наблюдалось. Пациенты с манифестацией ПГПТ в молодом возрасте демонстрировали более раннее развитие НЭН ЖКТ и аденом гипофиза. Результаты исследования подчеркивают необходимость раннего генетического скрининга и индивидуального мониторинга пациентов с МЭН-1 и их родственников с целью своевременного выявления заболевания.

## Дополнительная информация

Источники финансирования. Исследование выполнено за счет средств гранта РНФ 24-15-00269 «Геномный, транскриптомный и иммуногистохимический профиль при первично множественном поражении околощитовидных желез».

Конфликт интересов. Авторы декларируют отсутствие явных и потенциальных конфликтов интересов, связанных с содержанием настоящей статьи.

Участие авторов. Все авторы одобрили финальную версию статьи перед публикацией, выразили согласие нести ответственность за все аспекты работы, подразумевающую надлежащее изучение и решение вопросов, связанных с точностью или добросовестностью любой части работы.
